# Supplementary material for: Structural insights into C3 convertase activity of the classical pathway of complement
Source: Nat Commun. 2025 Dec 18;17:993. doi: 10.1038/s41467-025-67730-4 (PMC12847986; doi:10.1038/s41467-025-67730-4)

Structural insights into C3 convertase activity of the classical pathway of complement

Karla I. De la O Becerra[1], T. Harma C. Brondijk[1], Itziar Serna Martin[1,2], Piet Gros[1*]

[1] Structural Biochemistry, Bijvoet Centre for Biomolecular Research, Dept. of Chemistry, Faculty of Science, Utrecht University, Utrecht, The Netherlands

[2] Present address: Thermo Fisher Scientific, Electron microscopy division, Eindhoven, The Netherlands

* corresponding author: p.gros@uu.nl

Supplementary Figures

Supplementary Fig. 1

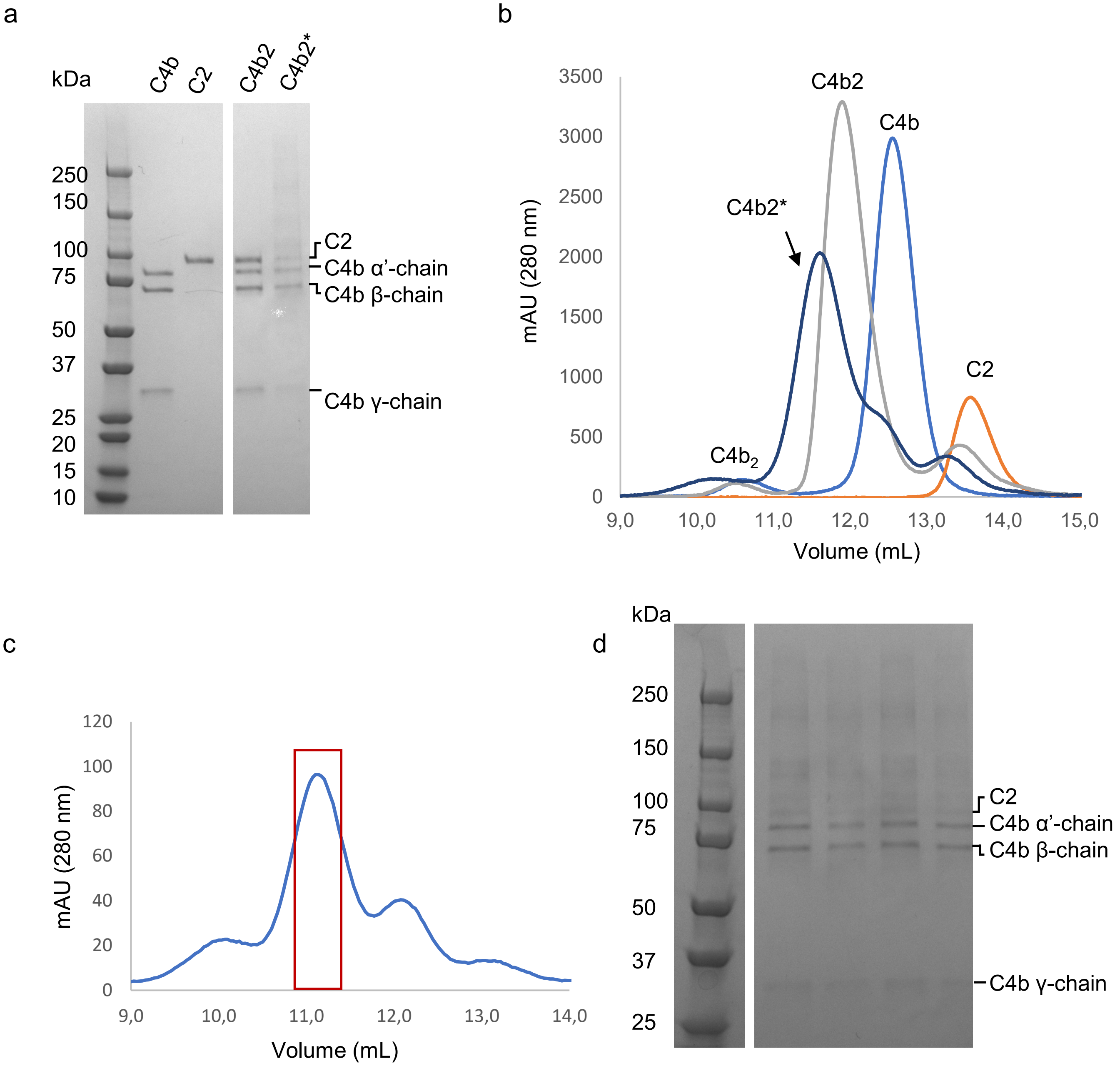

Supplementary Fig. 1. **Purification and cross-linking of proconvertase C4b2. a** SDS-PAGE gel and **b** analytic SEC of cross-linked C4b2 (C4b2*) with controls C4b, C2 and C4b2. **c** SEC of C4b2* with fractions selected for cryo-EM outlined in red and **d** SDS-PAGE gel of the selected fractions.

Supplementary Fig. 2

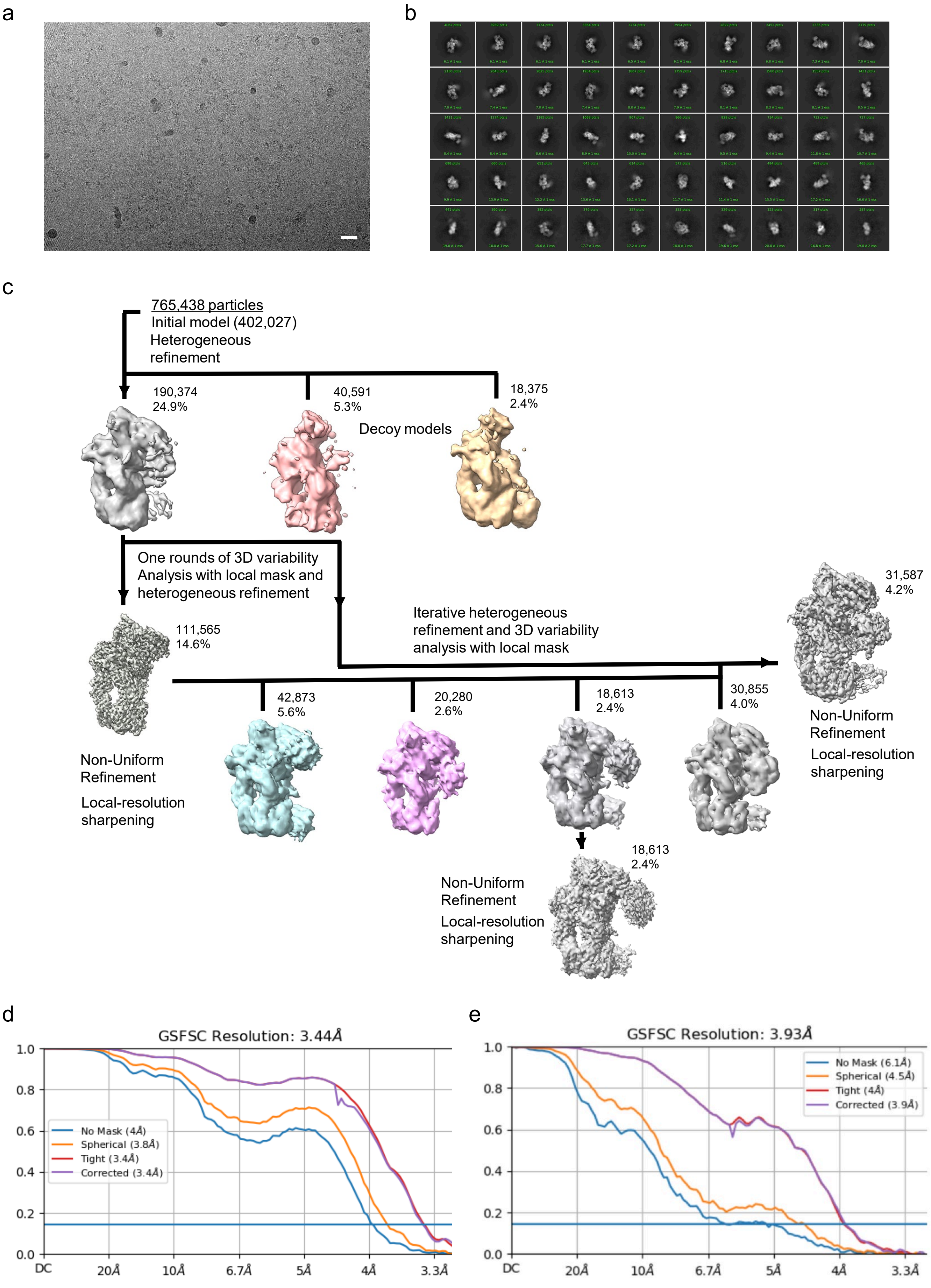

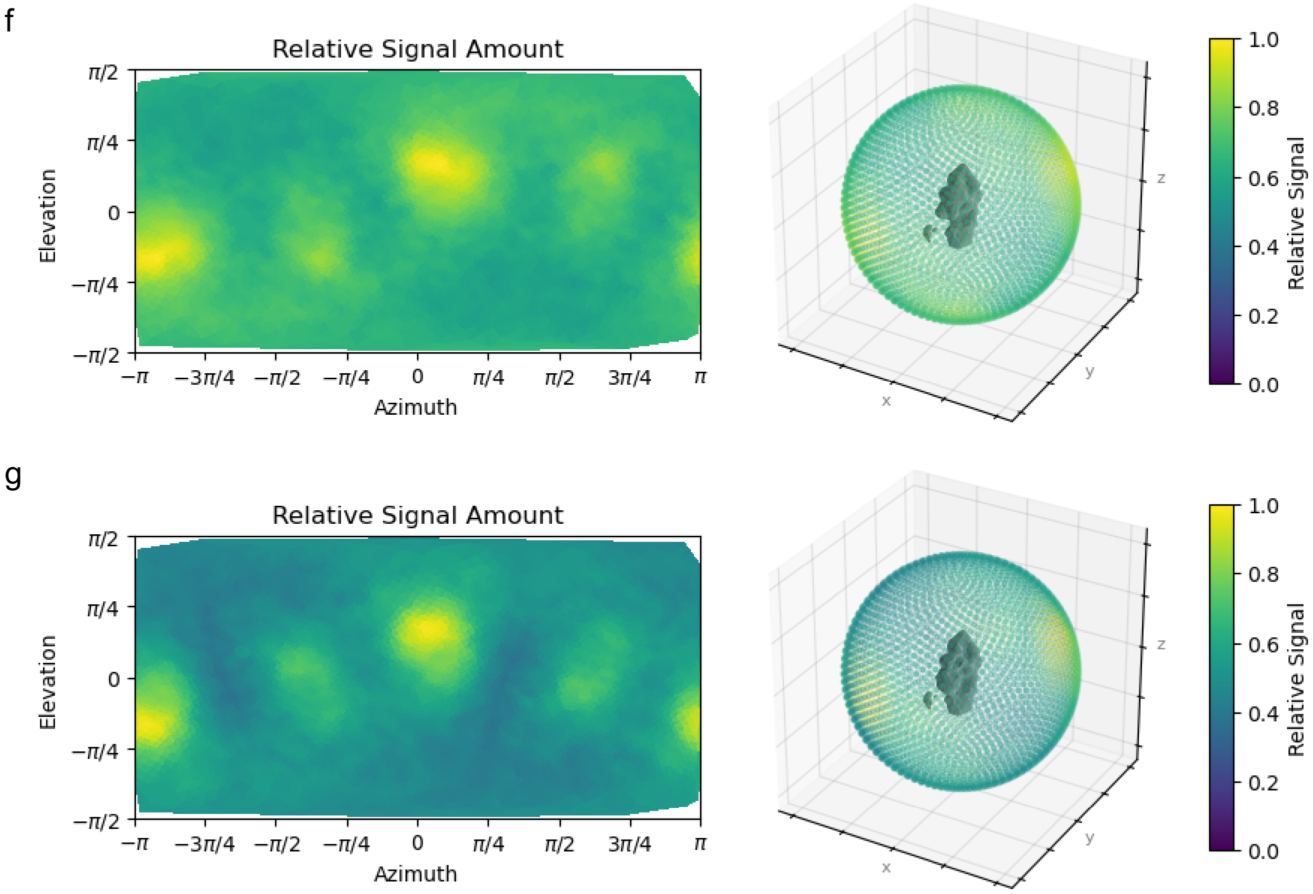

Supplementary Fig. 2. **Cryo-EM image processing of the proconvertase C4b2 dataset. a** Micrograph of cross-linked C4b2 particles in vitreous ice with a scale-bar length of 20 nm. **b** Selected 2D-class averages of the complex generated in cryoSPARC. **c** Cryo-EM processing workflow for C4b2 reconstructuin and refinement, including additional rounds of 3D variability analysis that reveals alternative SP domain conformations. **d-e** Gold-standard (0.143) Fourier shell correlation curves for the refined C4b2 maps at 3.5 Å and 3.9 Å resolution, computed using unmasked (blue), spherical mask (orange), tight mask (red), and mask-corrected (purple) FSC calculations. **f-g** Relative signal-amount analysis for the 3.5 Å and 3.9 Å resolution C4b2 reconstruction, respectively, showing the 2D angular heatmap (left) and corresponding 3D spherical projection (right). These plots depict the distribution of viewing-direction signal and highlight regions of preferred particle orientations.

Supplementary Fig. 3

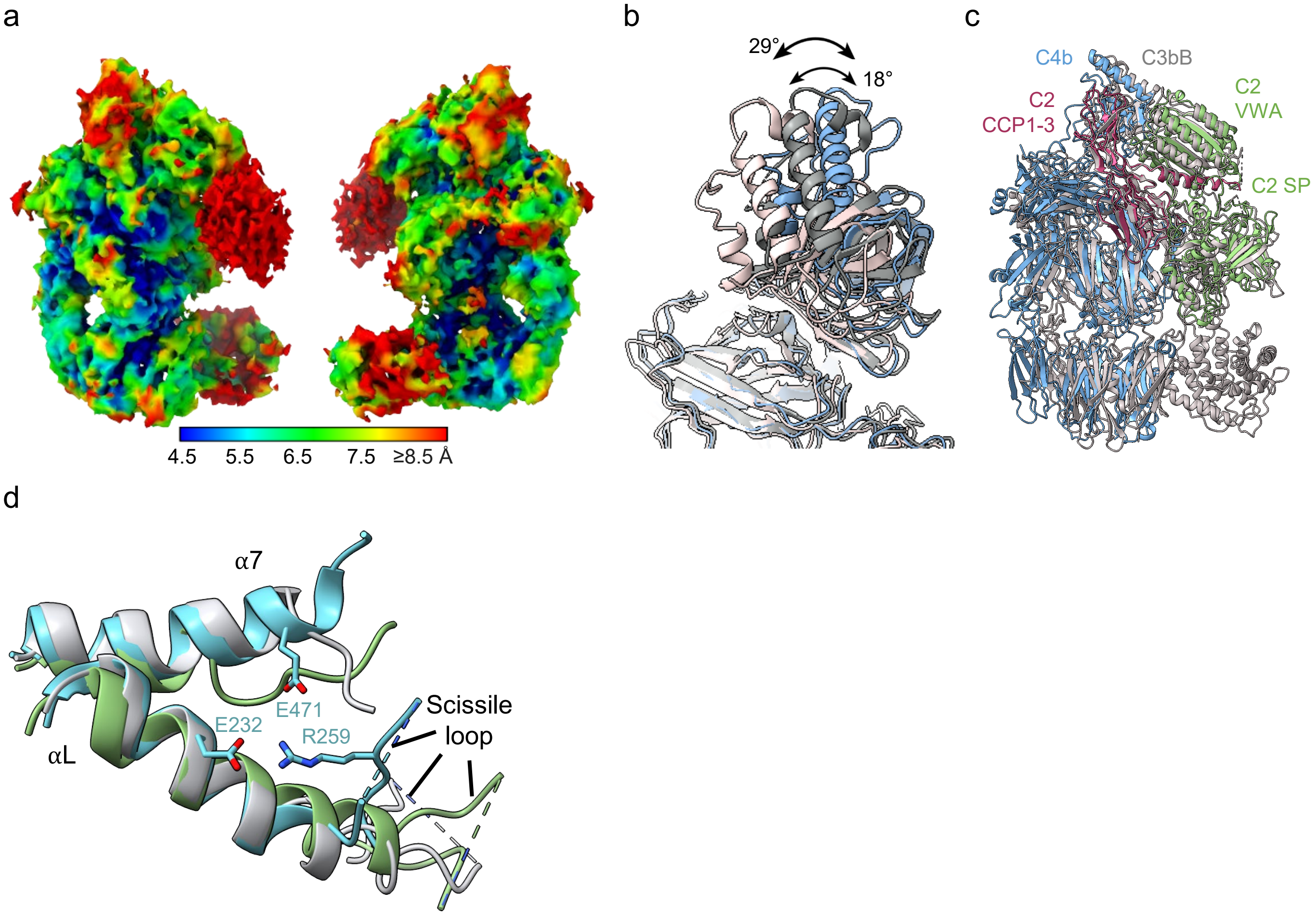

Supplementary Fig. 3. **Proconvertase C4b2. a** Cryo-EM density map of C4b2 at 4.2-Å resolution, reconstructed from a subset of particles selected via 3D variability analysis in CryoSPARC. The SP domain appears partially unresolved and displaced. The map is coloured by local resolution as indicated by the bar below. **b** Superposition free C4b (grey, PDB 5JPN) and C4b from the C4b-hC4Nb8 nanobody complex (light pink, PDB 6YSQ), onto C4b of the C4b2 complex (blue). **c** Superposition of C3bB (grey, PDB 2XWJ) on C4b2 with C4b in blue, C2 CCP1-2 and αL in purple and C2 VWA-SP in green. **d** Superposition of VWA domains from C4b2 (green), C3bB (grey), and CVF-B (cyan, PDB 3HRZ), showing helices αL and α7. Scissile loops in C4b2 and C3bB are exposed and disordered and exposed, while in CVF-B the FB P1-residue Arg259 is buried and interacts with αL (Glu232) and α7 (Glu471).

Supplementary Fig. 4

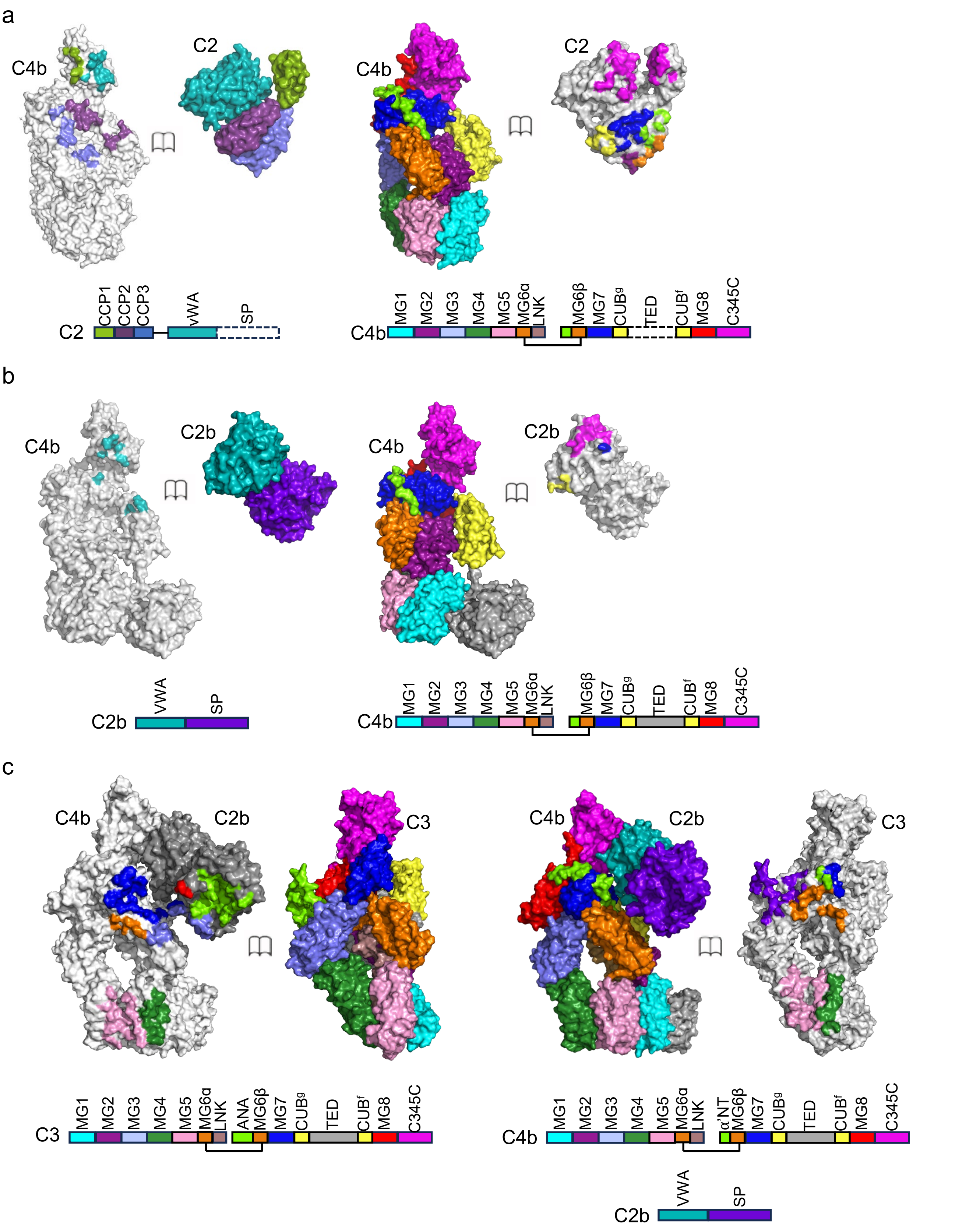

Supplementary Fig. 4**. Contact footprints of C4b2, C4b2b and C4b2bC3.** Open-book view of the contact footprints of **a** C4b2, **b** C4b2b and **c** C4b2b-C3. Domain coloring as indicated by the bar diagrams (domains missing in the model are indicated by dashed white bars).

Supplementary Fig. 5

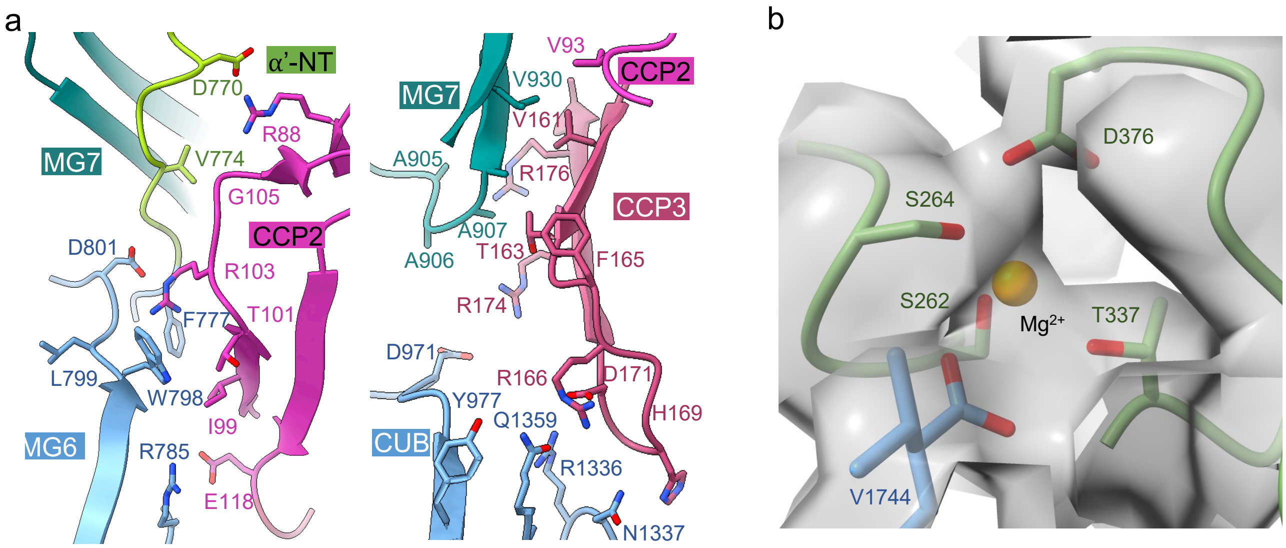

Supplementary Fig. 5. **Interactions between C4b and C2 in C4b2. a** Interactions between C4b MG6 (light blue), MG7 (blue-green) and α'N-terminal (α'NT, green) with C2 CCP2 (purple) (left panel) and C4b MG7 (blue-green) and CUB (blue) with C2 CCP2 (purple) and CCP3 (red) (right panel). **b** Cryo-EM density map shown in semi-transparent grey of the metal-ion dependent adhesion site (MIDAS) with C2 VWA interacting residues in green, the C-terminal tail of C4b γ-chain in blue and the magnesium ion as an orange sphere.

Supplementary Fig. 6

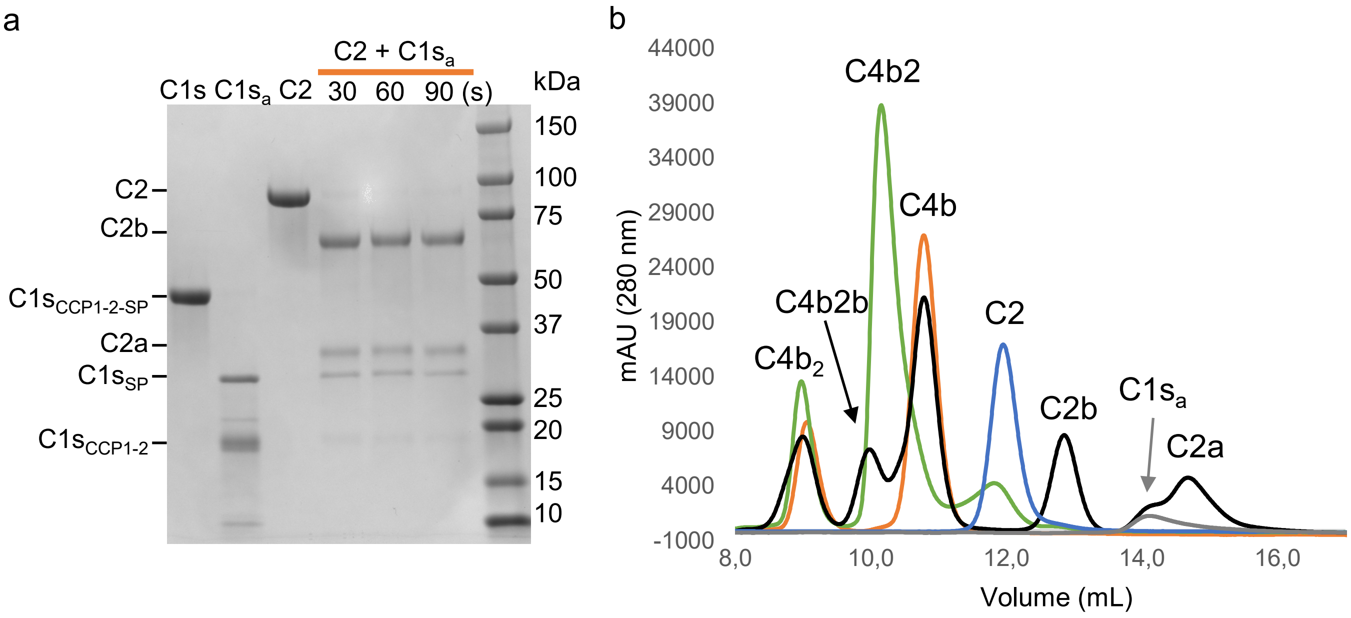

Supplementary Fig. 6. **Convertase formation. a** SDS-PAGE analysis of C2(S679A) cleavage into C2b and C2a fragments by enterokinase-activated C1s$_{CCP1-2-SP}$ (C1s$_a$). Lanes 1-3 show reference proteins C1s$_{CCP1-2-SP}$ (1 µg), C1s$_a$ (0.5 µg), and C2(S679A) (1 µg). Lanes 4-6 show cleavage of C2(S679A) by C1s$_a$ after 30, 60, and 90 seconds at room temperature, with 0.5 µg C2(S679A) and 0.05 µg C1s$_a$. **b** Analytical SEC profiles of proconvertase (green) and convertase (black) formation with C4b (orange), C2 (blue) and C1s$_a$ (gray) as reference.

Supplementary Fig. 7

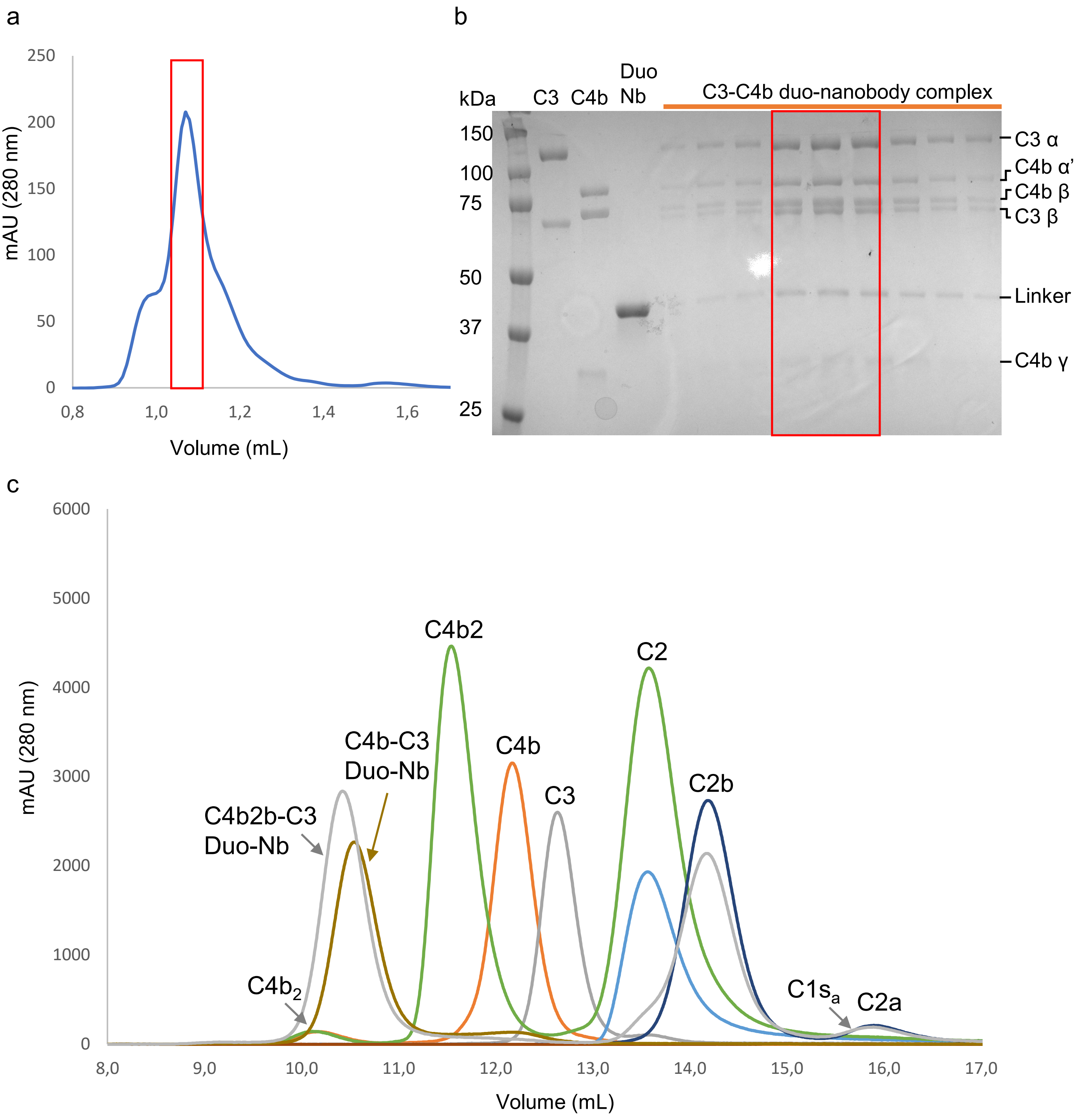

Supplementary Fig. 7. **Formation and purification of convertase-substrate complex C4b2bC3. a** SEC purification of anti-C4b and anti-C3 duo-nanobody complex with selected fractions outlined in red. **b** SDS-PAGE gel of selected fractions outlined in red and C4b, C3 and duo-nanobody linker as reference. **c** Analytical SEC of convertase-substrate C4b2b-C3 formation (grey) with activated $C1s_{CCP1-2-SP}$ ($C1s_a$), C2(S679A), C2b(S679A), C3, C4b, C4b2 and duo-nanobody as reference.

Supplementary Fig. 8

a

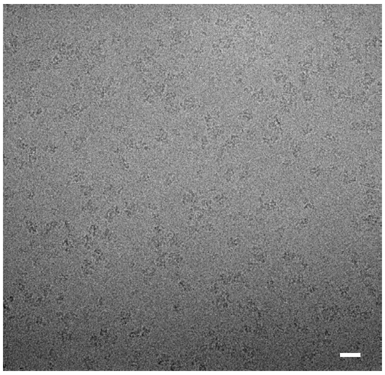

b

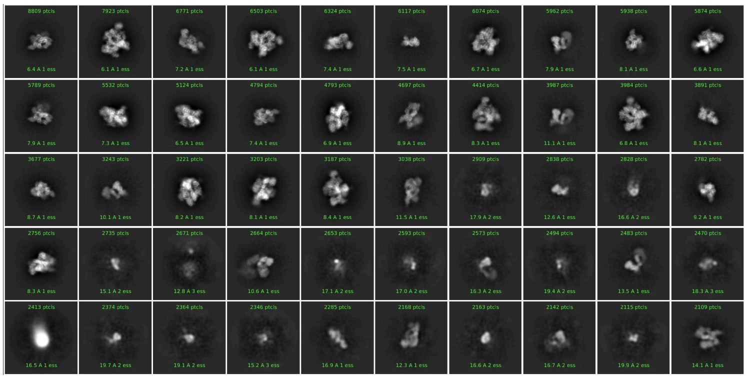

c

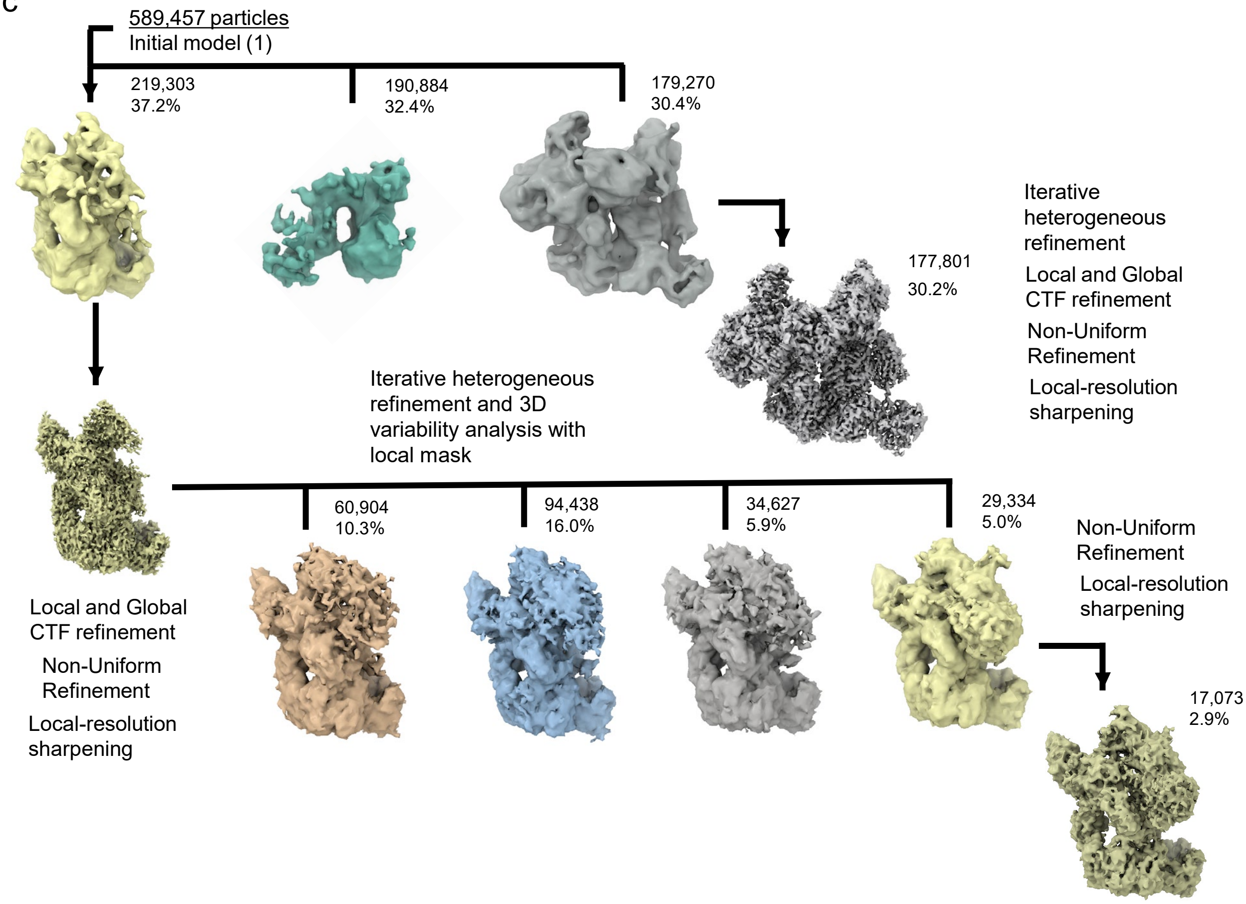

d

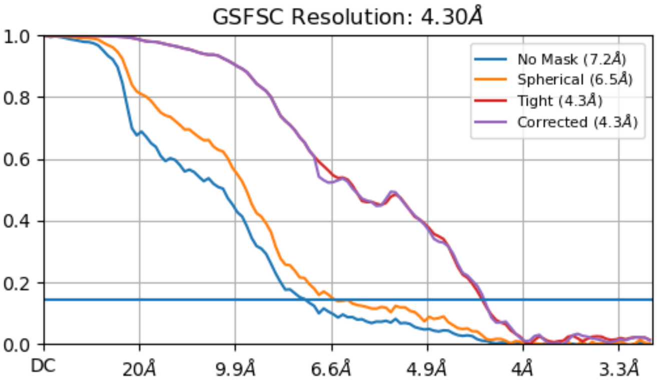

e

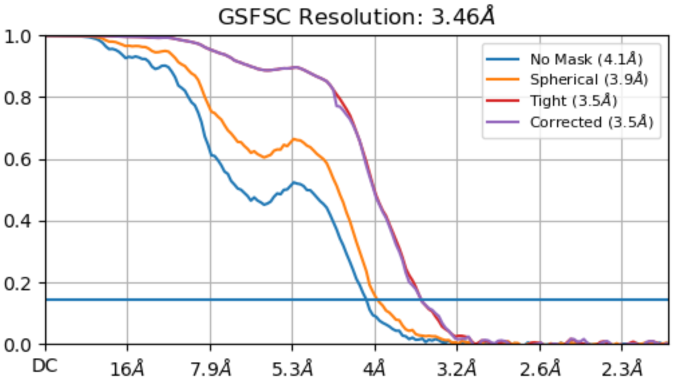

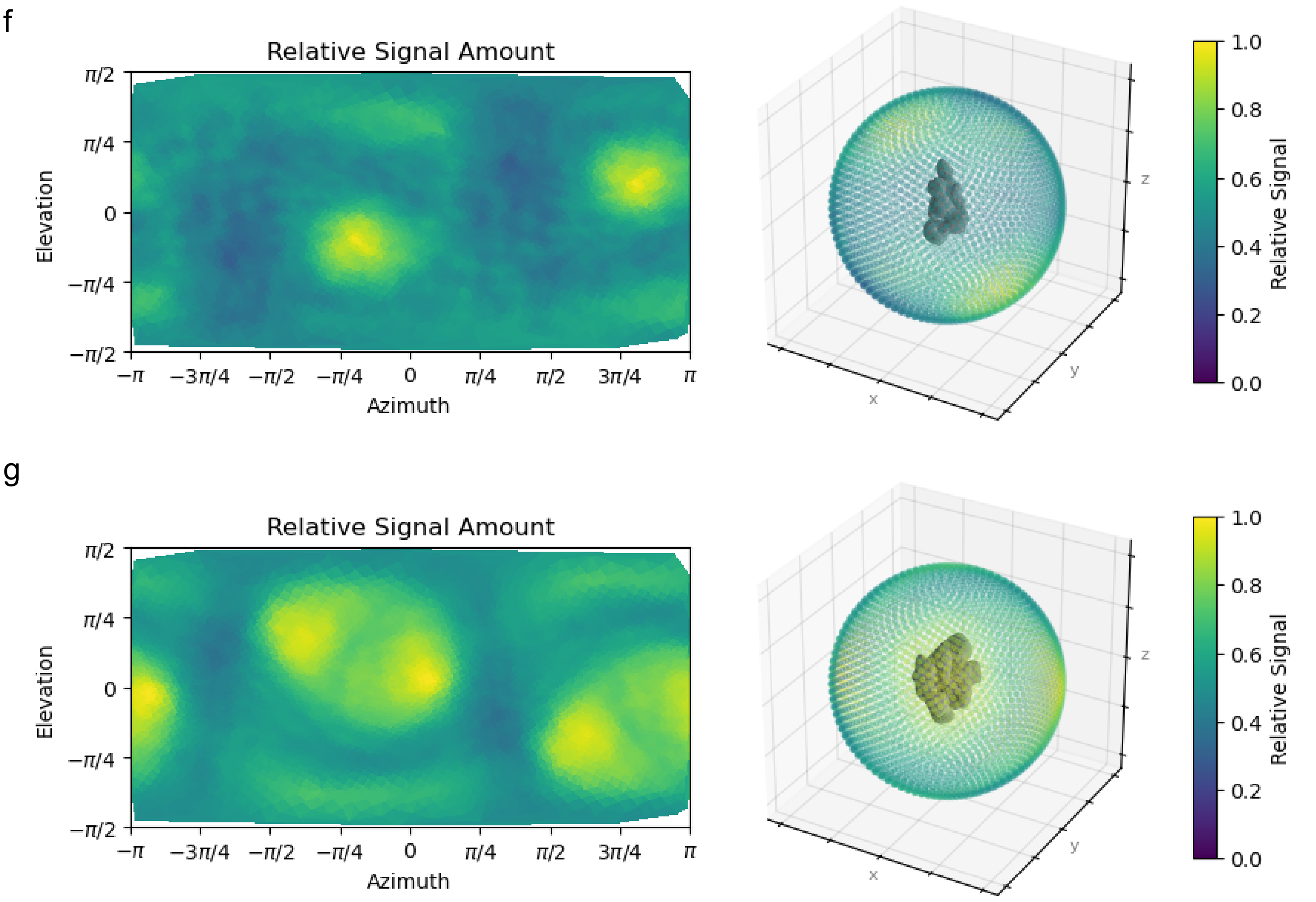

Supplementary Fig. 8. **Cryo-EM image processing of convertase C4b2b and convertase-substrate complex C4b2b-C3. a** Micrograph of C4b2b and C4b2b-C3 particles in vitreous ice with a scale bar of 20 nm. **b** Selected 2D-class averages of the complexes generated in cryoSPARC. **c** Cryo-EM processing workflow for Proteins C4b2b and C4b2b-C3, including extended 3D variability analysis for protein complex C4b2b to resolve C2b domains. **d-e** Gold-standard (0.143) Fourier shell correlation curves for the refined C4b2b and C4b2b-C3 reconstructions, respectively, computed from unmasked (blue), with a spherical mask (orange), tight mask (red), and corrected (purple). **f-g** Relative signal-amount analysis for the C4b2b and the C4b2b-C3 reconstruction, respectively, showing the 2D angular heatmap (left) and corresponding 3D spherical projection (right). These plots depict the distribution of viewing-direction signal and highlight regions of preferred particle orientations.

Supplementary Fig. 9

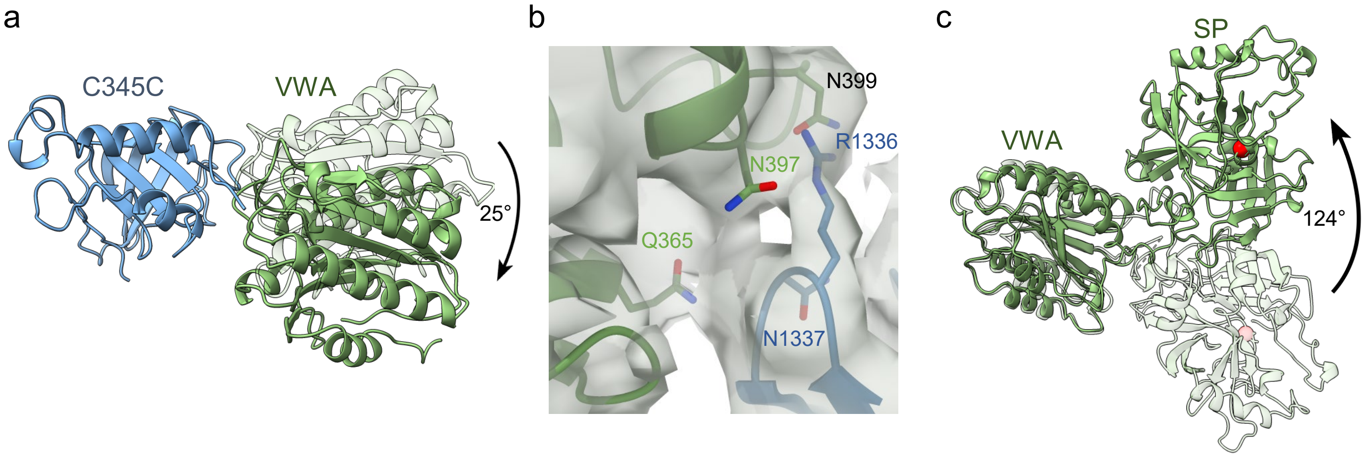

Supplementary Fig. 9. **Convertase, C4b2b. a** Superposition of C4b C345C domain (blue) of C4b2b and C4b2, with domain VWA shown in solid and transparent green, respectively, showing a 25° rotation. **b** Cryo-EM density map shown in semi-transparent grey; showing contact point of C4b CUB and C2b VWA. **c** Superposition of VWA domain of C4b2b and C4b2, with C2b VWA-SP shown in solid green and C2 VWA-SP in transparent green. SP domain rotates 124° repositioning and reorienting the enzyme active site. Cα of S679A is indicated by red spheres for both proteins.

Supplementary Fig. 10

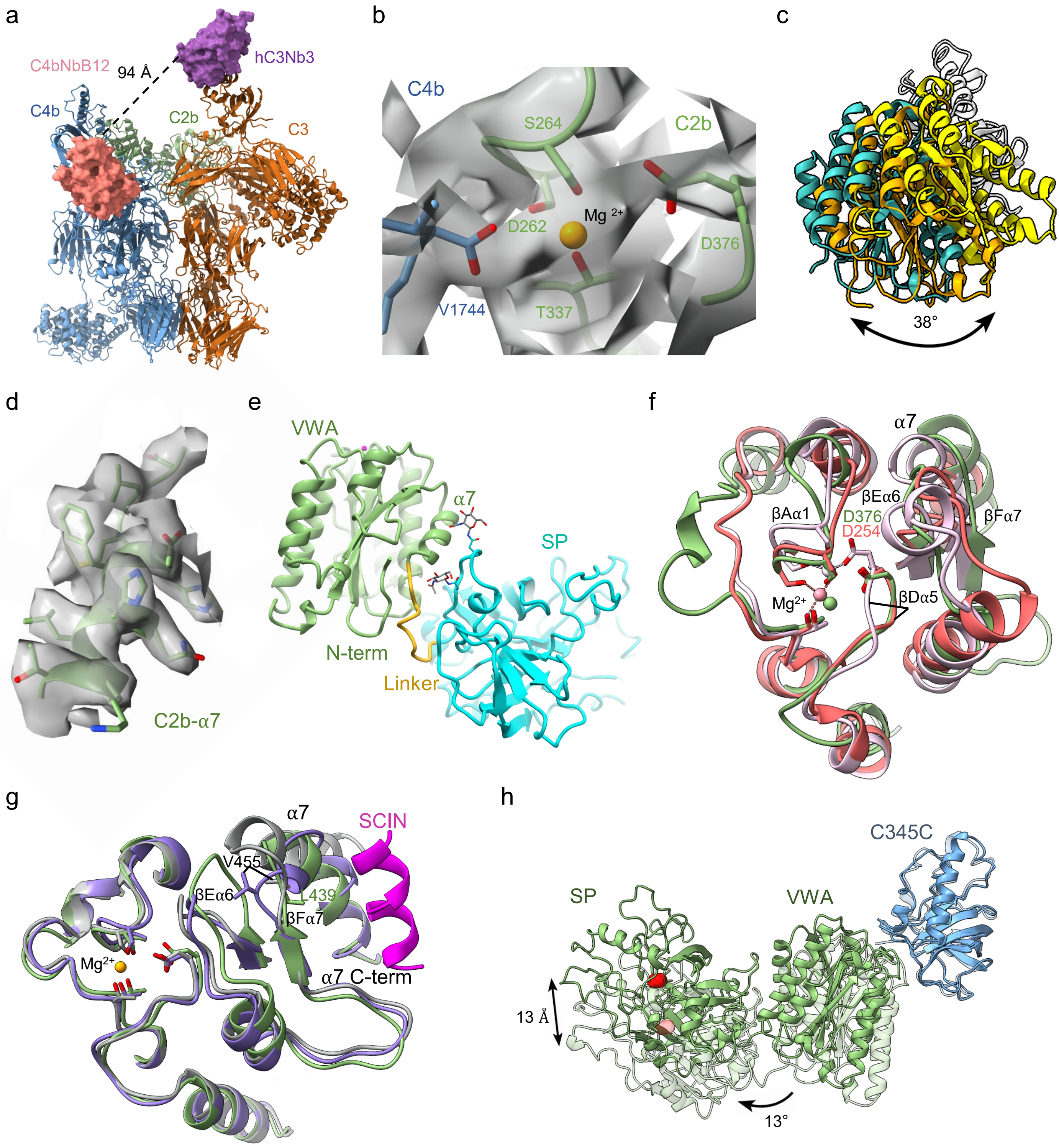

Supplementary Fig. 10. **Convertase-substrate C4b2b-C3.** **a** C4b2b-C3 shown in cartoon representation with C4b (blue), C2b (green) and C3 (orange). Anti-C4b nanobody C4bB12 and anti-C3 nanobody hC3Nb3 are shown in surface representation in pink and purple, respectively. The black dotted line indicates the minimum distance between the C and N-terminus of anti-C4b and anti-C3 duo-nanobody, resp. **b** Cryo-EM density map shown in semi-transparent grey of the MIDAS site with C2b residues shown in green, C-terminal tail of C4b in blue and Mg^{2+} in orange. **c** VWA domains of C4b2 (yellow), C4b2b (orange) and C4b2b-C3 (light blue) after superposing their C4b C343C domains. **d** Cryo-EM density map shown in semi-transparent grey of the a7 helix (green) of C2b in C4b2b-C3 complex. **e** C2b of C4b2b-C3 in cartoon representation with VWA (green), VWA-SP linker (orange) and SP (cyan) and modelled N-glycosylations (grey). **f** Superposition of integrin a2 I domain, in its unbound (light pink, PDB 1AOX), and ligand-bound state (dark pink, PDB 1DZI) onto C2b VWA (green) of C4b2b-C3; corresponding Mg^{2+} ions shown in respective colours. **g** FB VWA domains of FB from C3bB (grey, PDB 2XWJ) and Bb from $(C3bBb\text{-}SCIN)_2$ (purple, PDB 2WIN) with its interaction with SCIN (pink) superimposed onto C2b VWA (green) of C4b2b-C3 complex and its Mg^{2+} (orange). **h** C2b VWA-SP in C4b2b-C3 and C4b2b in solid and transparent green, respectively, after superposing their C4b C345C. C2b rotates 13° from free to substrate-bound convertase yielding displacement of S679A Ca by 13 Å.

Supplementary Fig. 11

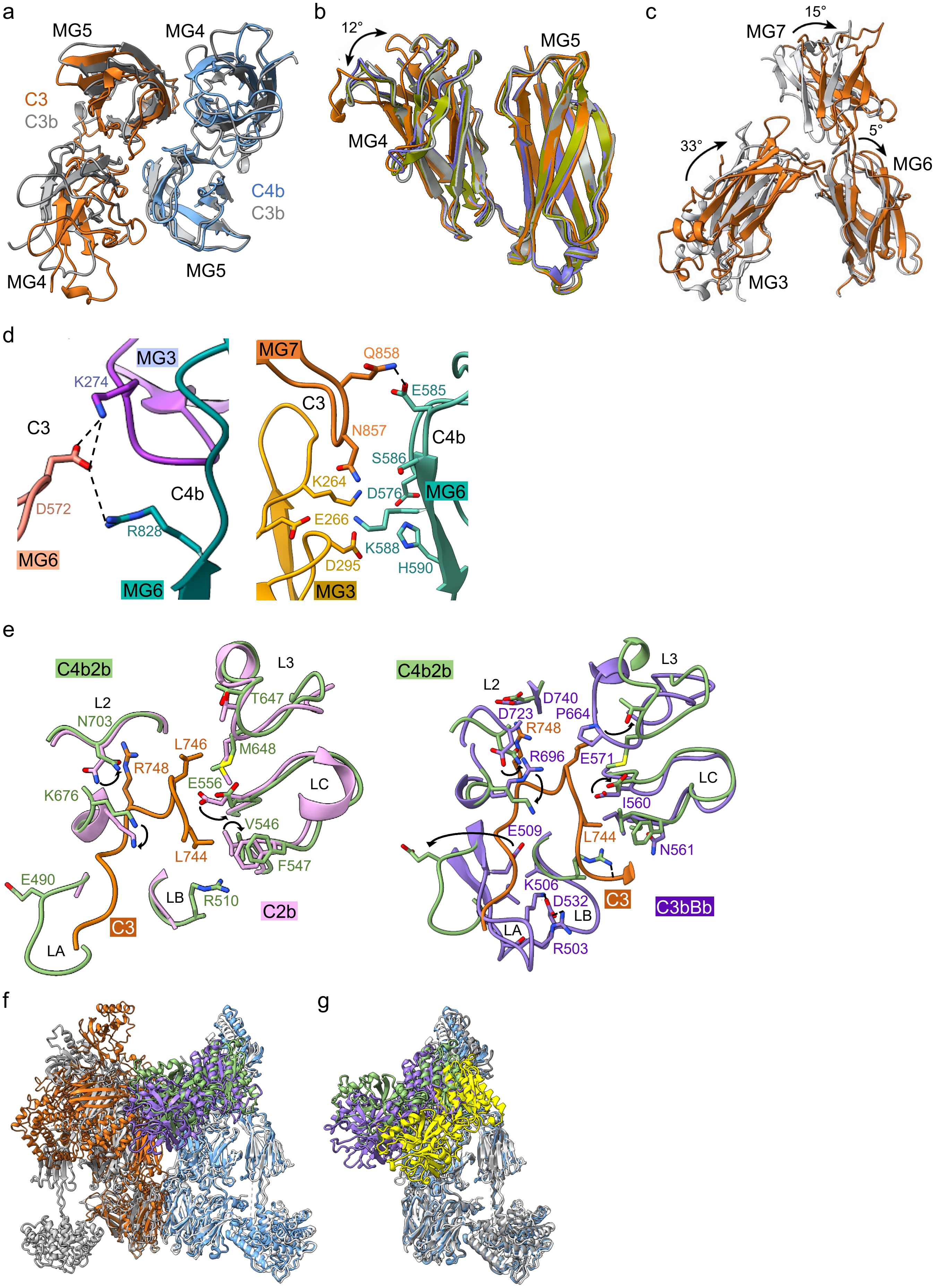

Supplementary Fig. 11. **Interactions between C3 and C4b2 in C4b2-C3**. **a** Cartoon representations of superposed C3b MG4-5 of $(C3bBb\text{-}SCIN)_2$ (grey, PDB 2WIN), C4b MG4-5 (blue) and C3 MG-5 (orange) of C4b2b-C3. **b** Superposition of MG4-5 of free C3 (grey, PDB 2A73), free C3b (green, 5FO7), C3b in $(C3bBb\text{-}SCIN)_2$ (purple) and C3 (orange) in C4b2b-C3. **c** C3 domains MG3, 6 and 7 of free C3 (grey) and C3 in C4b2-C3 (orange) after superposing on their MG5 domains. **d** Interactions between C4b MG3 (purple) and MG6 (teal) with C3b MG3 (yellow), MG6 (light pink) and MG7 (orange) in C4b2-C3. **e** Superposition of SP domain of free C2b (pink, PDB 2ODP) (left panel) and of Bb SP from $(C3bBb\text{-}SCIN)_2$ (purple, PDB 2WIN) (right panel) onto C2b (green) with bound C3-scissile loop (orange) of the C4b2b-C3 complex. **f** C4b2b-C3 (C4b in light blue, C2b in green and C3 in orange) and C3bBb-C3b (C3bBb, withC3b in white and Bb in purple, and C3b on substrate position in gray) taken from $(C3bBb\text{-}SCIN)_2$ (PDB 2WIN) superposed on the convertase C3b and C4b b-chains. **g** C3bBb of $(C3bBb\text{-}SCIN)_2$ (C3b in white and Bb in purple), free C4b2b (C4b in grey and C2b in yellow) and substrate-bound convertase C4b2b of C4b2b-C3 (C4b in light blue, C2b in green) superposed on their C3b or C4b β-chains.

Uncropped gel from Supplementary Fig. 1a

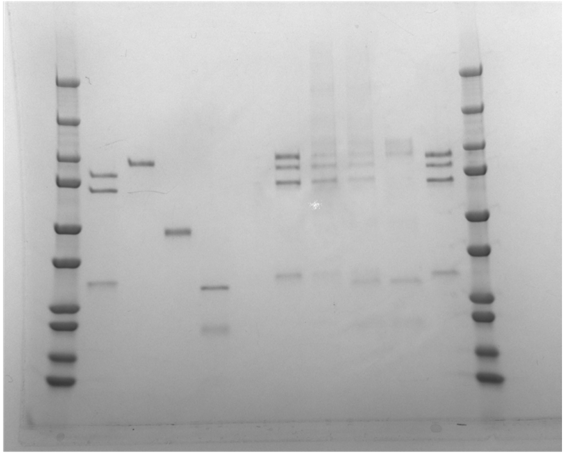

Uncropped gel from Supplementary Fig. 1d

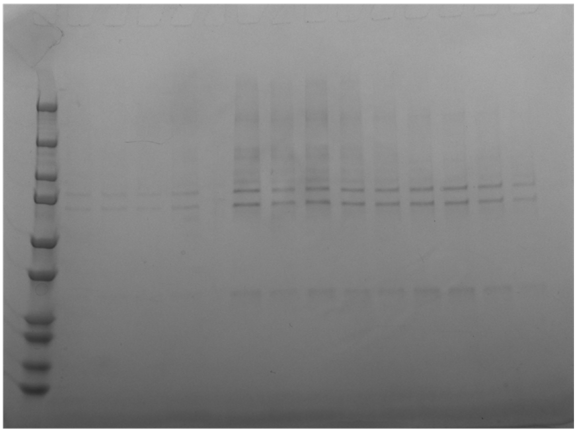

Uncropped gel from Supplementary Fig. 6a

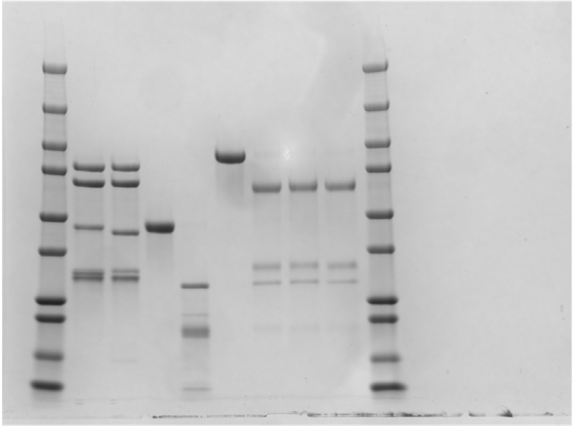

Uncropped gel from Supplementary Fig. 7b

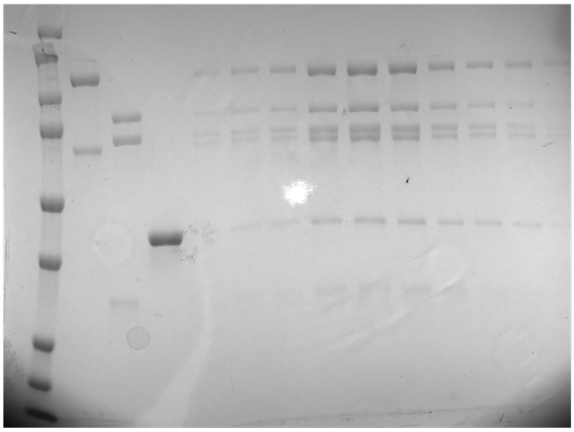

Supplement: Supplementary file 1 — Supplementary Information [file 41467_2025_67730_MOESM1_ESM.pdf]
