# Supplementary material for: Structural insights into C3 convertase activity of the classical pathway of complement
Source: Nat Commun. 2025 Dec 18;17:993. doi: 10.1038/s41467-025-67730-4 (PMC12847986; doi:10.1038/s41467-025-67730-4)
Supplement: Supplementary file 2 — Reporting Summary [file 41467_2025_67730_MOESM2_ESM.pdf]

nature portfolio

Corresponding author(s): Piet Gros

Last updated by author(s): 05/12/2025

Reporting Summary

Nature Portfolio wishes to improve the reproducibility of the work that we publish. This form provides structure for consistency and transparency in reporting. For further information on Nature Portfolio policies, see our Editorial Policies and the Editorial Policy Checklist.

Please do not complete any field with "not applicable" or n/a. Refer to the help text for what text to use if an item is not relevant to your study.
For final submission: please carefully check your responses for accuracy; you will not be able to make changes later.

Statistics

For all statistical analyses, confirm that the following items are present in the figure legend, table legend, main text, or Methods section.

n/a	Confirmed	
☐	☒	The exact sample size (*n*) for each experimental group/condition, given as a discrete number and unit of measurement
☒	☐	A statement on whether measurements were taken from distinct samples or whether the same sample was measured repeatedly
☒	☐	The statistical test(s) used AND whether they are one- or two-sided<br>*Only common tests should be described solely by name; describe more complex techniques in the Methods section.*
☒	☐	A description of all covariates tested
☒	☐	A description of any assumptions or corrections, such as tests of normality and adjustment for multiple comparisons
☐	☒	A full description of the statistical parameters including central tendency (e.g. means) or other basic estimates (e.g. regression coefficient) AND variation (e.g. standard deviation) or associated estimates of uncertainty (e.g. confidence intervals)
☒	☐	For null hypothesis testing, the test statistic (e.g. *F*, *t*, *r*) with confidence intervals, effect sizes, degrees of freedom and *P* value noted<br>*Give P values as exact values whenever suitable.*
☒	☐	For Bayesian analysis, information on the choice of priors and Markov chain Monte Carlo settings
☒	☐	For hierarchical and complex designs, identification of the appropriate level for tests and full reporting of outcomes
☒	☐	Estimates of effect sizes (e.g. Cohen's *d*, Pearson's *r*), indicating how they were calculated

Our web collection on statistics for biologists contains articles on many of the points above.

Software and code

Policy information about availability of computer code

Data collection	Proconvertase C4b2: EPU-2.10.00, C4b2b/C4b2b-C3: EPU-2.9.00
Data analysis	Cryo-EM data processing: C4b2: Cryosparc v3.2/3, C4b2b/C4b2b-C3: Cryosparc v4.2.0/4<br>Model building and refinement: Phenix v2.0-5793, Coot v0.9.8.94 and ChimeraX v 1.8/1.9

For manuscripts utilizing custom algorithms or software that are central to the research but not yet described in published literature, software must be made available to editors and reviewers. We strongly encourage code deposition in a community repository (e.g. GitHub). See the Nature Portfolio guidelines for submitting code & software for further information.

Data

Policy information about availability of data

All manuscripts must include a data availability statement. This statement should provide the following information, where applicable:

- Accession codes, unique identifiers, or web links for publicly available datasets
- A description of any restrictions on data availability
- For clinical datasets or third party data, please ensure that the statement adheres to our policy

DATA AVAILABILITY
The model coordinates and cryo-EM density maps of the structures have been deposited in the Protein Data Bank (PDB) and the Electron Microscopy Data Bank

(EMDB) under the following accession numbers; PDB 9QJ5 [https://doi.org/10.2210/pdb9QJ5/pdb] and EMDB-53199 [https://www.ebi.ac.uk/pdbe/entry/emdb/EMD-53199] for C4b2 (no SP), PDB 9QJ4 [https://doi.org/10.2210/pdb9QJ4/pdb] and EMD-53198 [https://www.ebi.ac.uk/pdbe/entry/emdb/EMD-53198] for C4b2, PDB 9QPY [https://doi.org/10.2210/pdb9QPY/pdb] and EMD-53288 [https://www.ebi.ac.uk/pdbe/entry/emdb/EMD-53288] for C4b2b, and PDB 9QK2 [https://doi.org/10.2210/pdb9QK2/pdb] and EMD-53217 [https://www.ebi.ac.uk/pdbe/entry/emdb/EMD-53217] for the C4b2b-C3 complex. The EMDB depositions include unfiltered-half maps, non-sharpened unmasked maps and sharpened masked maps. The source data underlying supplementary Figures 1b-c,6b and 7a,c are provided as a Source Data file. As described in the Methods section, structural models used to initiate model building are Protein Data Bank entries 5JTW [https://doi.org/10.2210/pdb5JTW/pdb], 2I6Q [https://doi.org/10.2210/pdb2I6Q/pdb], 3ERB [https://doi.org/10.2210/pdb3ERB/pdb], 2ODP [https://doi.org/10.2210/pdb2ODP/pdb], 7B2Q [https://doi.org/10.2210/pdb7B2Q/pdb] and 2A73 [https://doi.org/10.2210/pdb2A73/pdb]

Research involving human participants, their data, or biological material

Policy information about studies with human participants or human data. See also policy information about sex, gender (identity/presentation), and sexual orientation and race, ethnicity and racism.

Reporting on sex and gender	not applicable
Reporting on race, ethnicity, or other socially relevant groupings	not applicable
Population characteristics	not applicable
Recruitment	not applicable
Ethics oversight	The work presented in this study complies with all relevant ethical regulations. The use of human blood from healthy volunteers was approved by the Medical Ethics Committee of the University Medical Center Utrecht (METC protocol 07-125/C approved on March 1, 2010). Donors provided written, informed consent in accordance with the Declaration of Helsinki.

Note that full information on the approval of the study protocol must also be provided in the manuscript.

Field-specific reporting

Please select the one below that is the best fit for your research. If you are not sure, read the appropriate sections before making your selection.

☒ Life sciences ☐ Behavioural & social sciences ☐ Ecological, evolutionary & environmental sciences

For a reference copy of the document with all sections, see nature.com/documents/nr-reporting-summary-flat.pdf

Life sciences study design

All studies must disclose on these points even when the disclosure is negative.

Sample size	Cryo-EM structures of the proconvertase (C4b2) were obtained from a single crosslinked preparation containing C2 and C4b, while the convertase and convertase-substrate complexes (C4b2b and C4b2b-C3) were derived from a single preparation containing C4b, C3, duobody linker, C2(S679A), and preactivated C1s (CCP1-CCP2-SP). For the C4b2 complexes, one cryo-EM grid yielded 4,662 micrographs, from which 765,438 particles were initially extracted. After standard curation and classification, 111,565 particles contributed to the overall C4b2 reconstruction, and 31,587 to the C4b2 SP-domain-containing structure. For C4b2b and C4b2b-C3, a single grid collected across two sessions (1,700 and 2,576 micrographs) produced 589,457 initial particles. After curation and classification, 17,073 particles were used for the C4b2b map and 177,801 for the C4b2b-C3 map. The number of micrographs collected was determined by available cryo-EM instrument time rather than predetermined statistical sample-size calculations.
Data exclusions	Data were excluded only as part of the standard cryo-EM image-processing workflow. Micrographs were removed during curation if they exhibited poor CTF fit, excessive motion, abnormal defocus values, thick or crystalline ice, or contamination. Particles were first picked broadly using reference-free algorithms. During subsequent 2D and 3D classification, particles were separated into data-driven classes. Poorly aligned particles, junk classes, and particles not contributing to stable 3D structures were excluded. No additional data were removed beyond these standard quality-control steps. All curation procedures are mentioned in the methods section and shown in the Supplementary Figures.
Replication	Protein purification and complex formation were repeated multiple times (>5) during method optimization. Final cryo-EM data collection for each structure was performed from a single purified sample, using one grid for C4b2 and one grid for the C4b2b and C4b2b–C3 complexes (both present within the same protein mixture).
Randomization	Randomization arises from the reference-free steps in the cryoSPARC workflow. Initial particle picking was performed without structural templates, and particles were clustered by unsupervised 2D classification using stochastic Expectation-Maximization, which groups images solely by similarity. For one dataset, high-quality 2D classes obtained after ab-initio reconstruction were subsequently used as templates for improved particle repicking; this step does not impose structural bias beyond the 2D views observed in the data. All datasets underwent automated 3D classification and refinement using data-driven optimization. Final particle subsets were selected only on the basis of class quality and reproducibility.
Blinding	Blinding is not applicable to cryo-EM single-particle analysis. However, initial particle picking is performed in a reference-free manner, and computational classification is automated, minimizing user bias.

Reporting for specific materials, systems and methods

We require information from authors about some types of materials, experimental systems and methods used in many studies. Here, indicate whether each material, system or method listed is relevant to your study. If you are not sure if a list item applies to your research, read the appropriate section before selecting a response.

Materials & experimental systems

n/a	Involved in the study
☐	☒ Antibodies
☐	☒ Eukaryotic cell lines
☒	☐ Palaeontology and archaeology
☒	☐ Animals and other organisms
☒	☐ Clinical data
☒	☐ Dual use research of concern
☒	☐ Plants

Methods

n/a	Involved in the study
☒	☐ ChIP-seq
☒	☐ Flow cytometry
☒	☐ MRI-based neuroimaging

Antibodies

Antibodies used

We engineerd a dual specific nanobody linker fusing an anti-C4b nanobody which was selected in-house and nanobody hC3Nb3

Validation

Selection and validation of the C4b nanobody is described in Karla I. De la O Becerra et al. Multifaceted Activities of Seven Nanobodies against Complement C4b. The Journal of Immunology ji2100647 (2022) doi:10.4049/JIMMUNOL.2100647 and for the hC3bNb3 in Pedersen, H. et al. A Complement C3–Specific Nanobody for Modulation of the Alternative Cascade Identifies the C-Terminal Domain of C3b as Functional in C5 Convertase Activity. The Journal of Immunology 205, ji2000752 (2020). C3 and the nanobody linker were mixed in a concentration of 5 micromolar each and the complex was further purified by SEC.

Eukaryotic cell lines

Policy information about cell lines and Sex and Gender in Research

Cell line source(s)

Human

Authentication

Protein production in human cell lines (HEK293-ES and HEK293-E+) was done by a commercial party, Immunoprecise Antibodies BV. We have no information on their validation procedures

Mycoplasma contamination

Protein production in human cell lines (HEK293-ES and HEK293-E+) was done by a commercial party, Immunoprecise Antibodies BV. We have no information on their validation procedures

Commonly misidentified lines
(See ICLAC register)

Name any commonly misidentified cell lines used in the study and provide a rationale for their use.

Plants

Seed stocks

N/A

Novel plant genotypes

N/A

Authentication

N/A

This checklist template is licensed under a Creative Commons Attribution 4.0 International License, which permits use, sharing, adaptation, distribution and reproduction in any medium or format, as long as you give appropriate credit to the original author(s) and the source, provide a link to the Creative Commons license, and indicate if changes were made. The images or other third party material in this article are included in the article's Creative Commons license, unless indicated otherwise in a credit line to the material. If material is not included in the article's Creative Commons license and your intended use is not permitted by statutory regulation or exceeds the permitted use, you will need to obtain permission directly from the copyright holder. To view a copy of this license, visit http://creativecommons.org/licenses/by/4.0/
